# Development and validation of the rheumatoid arthritis scale among the system of quality of life instruments for chronic diseases QLICD-RA (V2.0)

**DOI:** 10.1038/s41598-024-58910-1

**Published:** 2024-04-18

**Authors:** Zheng Yang, Guannan Bai, Haifeng Ding, Mingyang Chen, Tong Xie, Chonghua Wan

**Affiliations:** 1 Research Center for Quality of Life and Applied Psychology, Key Laboratory for Quality of Life and Psychological Assessment and Intervention, Guangdong Medical University, Dongguan, 523808 China; 2 Department of Child Health Care, Children’s Hospital Zhejiang University School of Medicine, National Clinical Research Center for Child Health, Hangzhou, 310052 China; 3 Department of Geriatrics, The First Dongguan Affiliated Hospital of Guangdong Medical University, Dongguan, 523808 China; 4 Guangdong Prison Central Hospital, Guangzhou, 510430 China; 5 The Affiliated Hospital of Guangdong Medical University, Zhanjiang, 524001 China

**Keywords:** Quality of life, Rheumatoid arthritis, Instrument, Validation, Medical research, Rheumatology

## Abstract

Rheumatoid Arthritis is a more serious threatening to people and suitable for QOL measurement. A few specific QOL instruments are available without considering Chinese culture. The present study was aimed to develop and validate the Rheumatoid Arthritis Scale among the System of Quality of Life Instruments for Chronic Diseases (QLICD-RA V2.0). The data collected from 379 patients with RA was used to evaluate the psychometric properties of the scale. The reliability was evaluated by the internal consistency Cronbach’s α, test–retest reliability Pearson correlation r and intra-class correlation (ICC). We evaluated the construct validity and criteria-related validity by correlation analysis and structural equation modeling. We compared the differences in scores of QLICD-RA before and after treatment and used the Standard Response Mean (SRM) to assess the responsiveness. The results showed that the internal consistency coefficient Cronbach’s α values were greater than 0.70. The correlations r and ICCs were greater than 0.80. The correlation analysis and structural equation modeling confirmed good construct validity and criterion-related validity. The SRM ranges from 0.07 to 0.27 for significant domains/facets. It concluded that QLICD-RA (2.0) is a reliable and valid instrument to measure QOL among patients with RA.

## Background

Rheumatoid arthritis (RA) is a chronic, systemic, inflammatory disease of unknown etiology. It affects 0.3–1% of population worldwide^1^. The progressive course of RA may result in deformity and destruction of bones and joints. If untreated or unresponsive to therapy, it can lead to functional disability (e.g. difficulties in conducting daily activities), impaired psychological and social functioning and premature death^2–4^. In China, RA is a leading cause of disability, and cause heavy burden for the patients, families and society. For instance, the annual cost of RA was estimated to be approximately $13.9–22.4 billion in China^5,6^.

In recent decades, the concept of quality of life (QoL) has been received an increasing attention in the evaluation of clinical and medical interventions. The World Health Organization (WHO) defines QOL as ‘a broad ranging concept incorporating in a complex way the person’s physical health, psychological state, level of independence, social relationships, person’s beliefs and their relationship to salient features of the environment”^7^. RA is a major cause of an impairment of patient’s QOL. The disability and symptoms (e.g. pain, stiffness, fatigue) related to RA has significant impacts on patient’s physical, psychological and social health^8–11^. Therefore, the assessment of QOL is an integral way to evaluate the impacts of this disease on patient’s health and wellbeing, as well as to evaluate the effectiveness of medical treatment or health interventions^9,12,13^.

Some instruments to assess QOL of RA patients have been developed. Examples are the Quality of Life-Rheumatoid Arthritis scale(QOL-RA)^14^, Rheumatoid Arthritis Quality of Life Questionnaire(RAQoL)^15^, the McMaster Toronto Arthritis Patient Preference Disability Questionnaire, the Cedars-Sinai Heath-Related Quality of Life in Rheumatoid Arthritis instrument (CSHQ-RA)^16,17^, Juvenile Arthritis Quality of Life Questionnaire (JAQQ)^18^, the Arthritis Impact Measurement Scales (AIMS)^19,20^ and the Rheumatoid Arthritis Quality of life Scale(RAQOL)^21,22^. The above-mentioned instruments were mostly developed and validated in industrialized counties, which showed a relatively good feasibility, validity and sensitive to change in QOL of RA patients. However, these instruments are not developed by the popular modular approach-a general/core module plus specific modules. A popular trend in the field of scale development has been establishment of the comprehensive measurement tool that can capture both similarities and differences among diseases. By creating a general module for a class of diseases and additional modules for individual-specific variations, researchers hope to provide a more accurate assessment of patients’ quality of life. For example, both the QLQs (Quality of Life Questionnaires) from EORTC (European Organization for Research and Treatment) and the FACIT (Functional Assessment of Chronic Illness Therapy) in USA for QOL assessments have been developed based on this modular approach^23,24^.

Besides, QOL is cultural dependence. In Chinese culture, the family relationship and kinship play very important roles in daily life. Food culture is also thought of highly, and thus good appetite, sleep, and energy are highly regarded in daily life. Taoism and traditional medicine focus on good temper and high spirit. This kind of culture dependence does not reflect in most QOL instruments in other languages^25,26^.

Considering these needs, we developed a QOL system Quality of Life Instruments for Chronic Diseases (QLICD), which combines a general module with disease-specific modules^25,26^. In the second edition of QLICD, a general module (QLICD-GM) which can be used for all chronic disease, and 34 specific modules tailored to different diseases such as hypertension, psoriasis, chronic gastritis etc. have been developed^27–29^. Each module is designed exclusively for the relevant disease, ensuring precise evaluations. As an example, the hypertension scale (QLICD-HY V2.0) was formed by combining the QLICD-GM (V2.0) and the specific module for hypertension^27^. Similarly, the Chronic Gastritis instrument (QLICD-CG V2.0) was formed by combining the QLICD-GM (V2.0) and the specific module for this disease^29^.

In regard to Rheumatoid arthritis, we developed the QLICD-RA(V2.0) under the system, which is a multidimensional, disease-specific, self-administered questionnaire applied to measure QOL of RA patients. This study is aimed to present the development and validation of the QLICD-RA in RA patient population, including the reliability, validity and responsiveness.

## Methods

### Establishment of the general module QLICD-GM(V2.0)

QLICD-GM (V2.0) was developed on the basis of the first edition^25^. In order to consider the clear hierarchy of the theoretical structure, the theoretical framework was proposed after several rounds of qualitative work including nominal group and focus group discussions, and also in-depth interviews to doctors and patients. In addition, in order to consider comprehensiveness, the structure is further refined to the sub-lateral (facets) level. The test version (beta version) has a relatively large number of items (36), with 13 being for physical and 13 for psychological functions and 10 for social functions. At the process of item screening, the pilot and pre-test data was used to select item by quantitative statistical procedures including variation analysis, correlation analysis, factor analysis, doctor’s importance ratings and patients’ importance ratings. Also the in-depth interviews on items for doctors and patients and several rounds of focus group discussions were carried out. After these quantitative and qualitative works, 7 items were deleted and the formal version of QLICD-GM (V2.0) was formed containing 10 facets and 29 items. After a two-year practical applications and evaluation again at large samples, removing the urination item and merging the will and personality facets, the modified formal version of QLICD-GM (V2.0) which includes 9 facets and 28 items was further revised in 2015, with 9 items being for physiological function, 11 items for psychological function and 8 items for social function^28,29^.

### Establishment of the specific module

Based on a comprehensive literature review and experts’ experience, the members of the research group independently proposed 53 non-repeating items which formed the alternative item pool. Similar to the general module, the theoretical framework and item screening were carried out by several rounds of qualitative work including focus group discussions and in-depth interviews to doctors and patients. Specifically, some of the less important items have been deleted and some items reflecting specific social and psychological functions have been added. For instance, we separated item 1 into swelling and pain; replaced item 13 “muscles” with “muscle atrophy”, added an item “feel dry mouth”. Eventually the initial specific module contains 42 items.

We conducted a pretest of this 42-item questionnaire among RA patients (n = 30) and medical staff (n = 26) in two hospitals in Kunming and Zhanjiang in order to evaluate whether all the items were sensitive, representative, and comprehensive. Besides 42 items, the questionnaires of RA patients and medical staff were different, focusing on cognitive Interview for the former and evaluation interview for the later. Similarly, quantitative statistical procedures of variation analysis, correlation analysis, doctor’s importance ratings and patients’ importance ratings, and also the in-depth interviews for doctors and patients were used to analyze and evaluate items. Results of the pretest have been discussed in two rounds by the research group members. After the first round of discussion, 22 items were deleted. Later on, in order decrease the response burden on patients, 5 items were deleted based on measurements for test version. Finally, the specific module with 15 items was formed, which classify into 3 facts of Limitation of activity(LOA), Complications(COM) and treatment side effects, and Joint pain and deformity(JPD).

**Figure 1:**
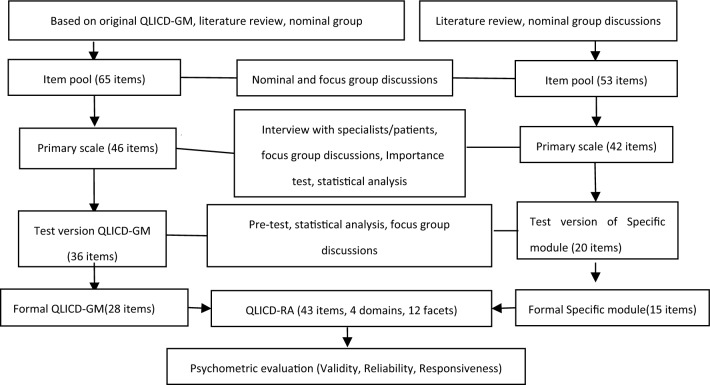
Steps towards development and validation procedure of QLICD-RA (V2.0).

The above steps to form the final version of QLICO-RA were presented in Fig. 1.

### Validation of the QLICD-RA

#### Data collection and scoring

The research protocol, along with the informed consent document, gained approval from the Institutional Review Boards (IRBs) at the investigators’ respective institutions and the associated hospital. In terms of sample size, according to our experience and estimation based on variation from 0–100 standardized scores^30,31^, 200 cases are enough for validation of the scale because of using sensitive statistical methods such as Pearson correlation analysis and paired-t tests.

We recruited 379 patients diagnosed with RA for our study, and these participants came from the Affiliated Hospital of Kunming Medical University and the Affiliated Hospital of Guangdong Medical University in China, and were screened by their treating physicians and the investigative team. The enrolled participants were capable of understanding and completing the questionnaires related to the various stages of treatment and volunteered to participate in this study.

All participants filled in the questionnaires of the QLICD-RA, the Chinese version of SF-36^32^ on the first day of admission to the hospital by themselves. Among them, 47 received the second measurements on the second day of hospitalization for test–retest reliability, and 223 measured again at the day before discharge for responsiveness. The investigators were checked the answers immediately to ensure the completeness of the answers each time. If missing values were found, the questionnaire would be returned to the patients to fill in the missing item.

Each item of QLICD-RA is based on a five-level scale, namely, not at all, a little bit, somewhat, quite a bit, and very much. The positively stated items directly obtain scores from 1 to 5 points and the negatively stated items are reversed. The domain and the overall scores are obtained by adding together the within-domain item scores. For comparison, all domains scores were linearly converted to a 0–100 scale using the formula: SS = (RS − Min) × 100/R, where SS, RS, Min and R represent the standardized score, raw score, minimum score, and range of scores, respectively. The higher score indicates better QOL.

#### Psychometrics analysis

To evaluate the internal consistency of our measurement tool, we conducted several statistical analyses. We calculated Cronbach’s alpha coefficients for each domain/facet of the scale, and item-to-domain score correlations using Pearson correlation coefficients. A Cronbach’s alpha coefficient exceeding 0.70 was considered indicative of good internal consistency, while an item-to-total score correlation exceeding 0.40 indicated good item internal consistency^33^.

In order to assess the test–retest reliability of the QLICD-RA instrument, we employed correlation coefficients (r) and intra-class correlation coefficients (ICCs). The threshold for test–retest reliability was defined as ICC 0.80.

We evaluated the convergent validity and discriminant validity, which also represented the construct validity. Pearson correlation analysis was applied to assess the correlation between the item scores and domain scores. The correlation coefficients were interpreted according to the following criteria^27,34^: (1) the convergent validity is supported when an item-domain correlation is greater than 0.40; (2) the discriminant validity is revealed when the correlation between the score of an individual item and the score of its designated domain is stronger than that between the score of this item and non-designated domains. We additionally evaluated the construct validity of the specific module and the general module respectively by confirmatory factor analysis using structural equation modeling, with the CFI (comparative fit index) and TLI (Tucker-Lewis index) greater than 0.90, RMSEA(root-mean-square error of approximation) less than 0.08 and SRMR(standardized root mean square residual) less than 0.10 reflecting a good fit of the model to the data^35,36^. Criterion-related validity was evaluated by calculation the correlation coefficients between domain scores of QLICD-RA and domain scores of Chinese SF-36 (the 36 item Short Form Health Survey)^32^.

Responsiveness was assessed through comparing the mean difference between the pre-treatment and post-treatment with effect size, SRM (standardized response mean).

All statistical analysis was done with SPSS (version 22.0) software.

### Ethical approval and consent to participate

The study protocol was approved by the Institutional Review Board (IRB) of the first affiliated hospital of Guangdong medical university (PJ2013037). The investigators explained the aims of the trial and the instrument to the patients and obtained informed consent from those patients who agreed to participate in the study and met with the inclusion criteria. A complete assurance was given that all information would be kept confidential. The right was given to the patients not to participate and to discontinue participation in the study with consideration /without penalty. The Declaration of Helsinki’s ethical guidelines were followed in the study.

## Results

### Socio-demographic characteristics of the participants

The socio-demographic characteristics of the participants were presented in Table 1. Among the 372 participants, around half were older than 50 years; 80% female; above 70% had moderate educational level (i.e. high school); around 90% were married and 97% had Han ethnicity. The diagnosis of most participants is typical RA and was at chronic stage. Around 35% participants had immunosupressor treatment, and 40% had both hormone and immunosuppresor treatment. 34% of participants had public insurance, while around half paid all the cost by themselves.

**Table 1:** Socio-demographic characteristics of the sample (n = 379).

Characteristics	N	%	Characteristics	N	%
Gender			Marital status		
Male	79	20.84	Married	331	87.34
Female	300	79.16	Others	48	12.66
Ethnic groups			Medical insurance		
Han	367	96.83	Self-paid	177	46.70
Others	12	3.17	Partly public insurance	73	19.26
Missing	0	0	Public insurance	129	34.04
			Missing	0	0
Age			Occupation		
< 30	40	10.55	Factory Worker	63	16.62
30–39	52	13.72	Farmer	142	37.47
40–49	88	23.21	Teacher	15	3.96
50–59	114	30.08	Officer/manager	34	8.97
≥ 60	85	22.43	Others	125	32.98
Missing	0	0			
Income#			Course		
Poor	119	31.40	Acute stage	47	12.40
Fair	245	64.64	Subacute stage	20	5.28
High	15	3.96	Chronic stage	166	43.80
Missing	0		Relief stabilization period	46	12.14
			Missing	100	26.39
Education			Treatments		
Primary school	84	22.16	Hormone (H)	9	2.37
High school	277	73.09	Immunosuppressor (I)	131	34.56
College or higher	18	4.75	Biologicals (B)	3	0.79
Missing	0	0	Neither (H + I)	51	13.46
			Both ( H + B)	27	7.12
			Both (H + I)	158	41.69

^#^This is evaluated by patients himself/herself according to their perceptions.

### Reliability

Table 2 shows the Cronbach’s α, test–retest reliability coefficients (correlation r and ICC) for domains and modules, as well as the overall instrument. The range of Cronbach’s α values was 0.77–0.94 at domains level, which was greater than 0.70. Forty three patients completed the questionnaires for test–retest reliability analysis. The correlation r ranged from 0.86 to 0.99. All the values of ICC were greater than 0.80.

**Table 2:** Reliability of the quality of life instrument QLICD-RA(V2.0) (n = 379 for α, and floor and ceiling effects, n = 47 for r, ICC).

Domains/facets	Internal consistency coefficient α	Test–retest reliability correlation *r*	ICC (95%CI)
Physical domain (PHD)	**0.77**	**0.98**	**0.98 (0.96–0.99)**
Basic physiologic functions (BPF)	0.53	0.97	0.96 (0.94–0.98)
Independence (IND)	0.89	0.98	0.98 (0.96–0.99)
Energy and discomfort (EAD)	0.61	0.86	0.86 (0.76–0.92)
Psychological domain (PSD)	**0.85**	**0.98**	**0.98 (0.96–0.99)**
Cognition (COG)	0.64	0.96	0.96 (0.93–0.98)
Emotion (EMO)	0.83	0.99	0.99 (0.98–0.99)
Will and personality (WIP)	0.58	0.89	0.89 (0.80–0.94)
Social domain (SOD)	**0.77**	**0.96**	**0.96 (0.93–0.98)**
Interpersonal communication (INC)	0.61	0.97	0.97 (0.95–0.98)
Social support and security (SSS)	0.57	0.95	0.95 (0.91–0.97)
Social role (SOR)	0.64	0.94	0.94 (0.90–0.97)
Sub-total (QLICD-GM)	**0.90**	**0.99**	**0.99 (0.97–0.99)**
Specific domain (SPD)	**0.92**	**0.98**	**0.98 (0.96–0.99)**
Limitation of activity(LOA)	0.88	0.99	0.99 (0.98–0.99)
Complications & side-effects(COM)	0.74	0.97	0.97 (0.95–0.98)
Joint pain and deformity(JPD)	0.88	0.97	0.97 (0.94–0.98)
Total (TOT)	**0.94**	**0.99**	**0.99 (0.98–0.99)**

*ICC* Intra-class correlation, *CI* Confidence interval.

The values at domain/overall level are in bold.

### Validity

#### Construct validity

Table 3 shows the correlation coefficients between items and domains of QLICD-RA. All correlation coefficients between the scores of items and their relevant domains were greater than 0.40, which indicates a good convergent validity. The correlation coefficient between the score of every item and the score of its designated domain was greater than that with its non-designated domains, except for the item ‘Attention’ (GPS1), which indicates good discriminant validity.

**Table 3:** Correlation coefficients r among items and domains of QLICD-RA(V2.0) (n = 379).

Code	Items brief description	Physical	Psychological	Social	The specific
GPH1	Appetite	0.51**	0.33**	0.29**	0.34**
GPH2	Sleep	0.49**	0.27**	0.23**	0.25**
GPH3	Sexual function	0.45**	0.23**	0.25**	0.28**
GPH4	Excrement	0.51**	0.24**	0.33**	0.20**
GPH5	Pain	0.60**	0.39**	0.28**	0.50**
GPH6	Daily activities	0.78**	0.29**	0.50**	0.57**
GPH7	Work	0.75**	0.30**	0.52**	0.52**
GPH8	Walk	0.71**	0.24**	0.43**	0.47**
GPH9	Fatigue	0.52**	0.49**	0.28**	0.32**
GPS1	Attention	0.57**	0.42**	0.49**	0.38**
GPS2	Memory deterioration	0.22**	0.51**	0.14**	0.23**
GPS3	Joy of life	0.34**	0.39**	0.34**	0.23**
GPS4	Restless	0.27**	0.68**	0.30**	0.36**
GPS5	Family burden	0.25**	0.70**	0.33**	0.31**
GPS6	State of health	0.30**	0.69**	0.31**	0.28**
GPS7	Depression	0.30**	0.70**	0.32**	0.36**
GPS8	Disappointment	0.31**	0.75**	0.37**	0.41**
GPS9	Fear	0.30**	0.75**	0.34**	0.33**
GPS10	Positive attitude	0.44**	0.52**	0.51**	0.36**
GPS11	Termagancy	0.29**	0.74**	0.34**	0.35**
GSO1	Social contact	0.56**	0.33**	0.70**	0.39**
GSO2	Family relationship	0.19**	0.12*	0.49**	0.12*
GSO3	Friend relationship	0.21**	0.08	0.51**	0.12*
GSO4	Family support	0.35**	0.26**	0.67**	0.29**
GSO5	Other people’s care	0.36**	0.28**	0.69**	0.25**
GSO6	Economic hardship	0.37**	0.57**	0.63**	0.41**
GSO7	Labor status	0.36**	0.54**	0.61**	0.42**
GSO8	Family role	0.45**	0.28**	0.68**	0.35**
RA1	Finger joint pain	0.50**	0.40**	0.32**	0.72**
RA2	Joint pain in the morning	0.43**	0.35**	0.26**	0.73**
RA3	Dysarthrasis	0.41**	0.32**	0.36**	0.71**
RA4	Arthralgia on exertion	0.50**	0.38**	0.34**	0.74**
RA5	Arthralgia if inactive	0.51**	0.41**	0.36**	0.76**
RA6	Amyotrophy	0.46**	0.38**	0.41**	0.72**
RA7	Siogren syndrome	0.32**	0.40**	0.35**	0.57**
RA8	Nausea	0.23**	0.37**	0.25**	0.45**
RA9	Dyspnea	0.23**	0.36**	0.22**	0.47**
RA10	Comb hair	0.49**	0.34**	0.35**	0.76**
RA11	Eat with chopsticks	0.49**	0.30**	0.39**	0.77**
RA12	Buckle the knot	0.49**	0.25**	0.34**	0.51**
RA13	Neck pain	0.45**	0.39**	0.35**	0.73**
RA14	Stoop	0.59**	0.31**	0.42**	0.79**
RA15	Get in and out bed	0.58**	0.34**	0.38**	0.72**

**There was a significant at the level of 0.01. *There was a significant at the level of 0.05.

**Figure 2:**
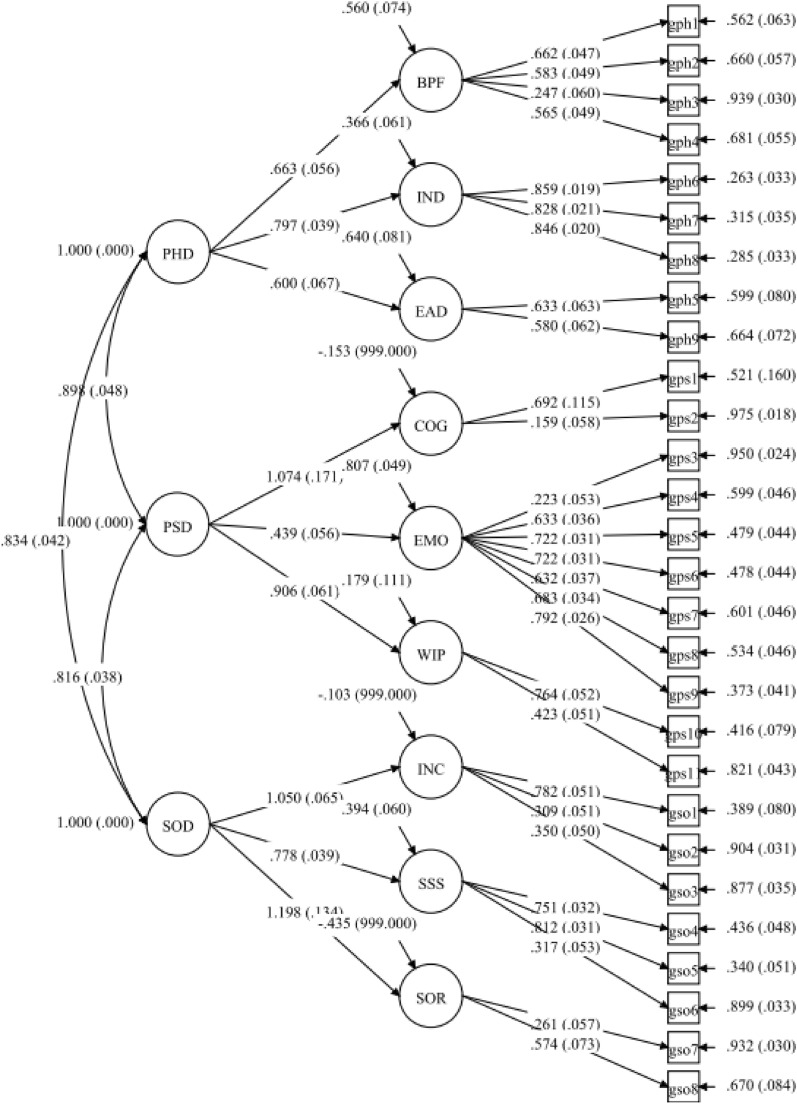
The structure of the general module of QLICD-RA by structural equation modeling.

Structural equation modeling showed that the structure of the general module of the QLICD-RA was roughly consistent with the conceptual theoretical construct (three domains, nine facets), with relatively not higher goodness of fit indicators: Chi-square ${{\varvec{\chi}}}^{2}$= 1059.817 (*P* < 0.001), df = 332, ${{\varvec{\chi}}}^{2}$/df = 3.192, Tucker–Lewis index (TLI) = 0.805, comparative fit index (CFI) = 0.829, root mean square error of approximation (RMSEA) = 0.076, standardized root mean square residual (SRMR) = 0.116. See Table 4 and Fig. 2 in detail.

**Table 4:** The results of SEM analysis on the general module of QLICD-RA (n = 379)*.

Domains	Facets	Items/standardized path coefficients
Physical function (PHD)	Basic physiologic functions (BPF)	GPH1/0.662 GPH2/0.583 GPH3/0.247 GPH4/0.565
	Independence (IND)	GPH6/0.859 GPH7/0.828 GPH8/0.846
	Energy and discomfort (EAD)	GPH5/0.633 GPH9/0.580
Psychological function (PSD)	Cognition (COG)	GPS1/0.692 GPS2/0.159
	Emotion (EMO)	GPS3/0.223 GPS4/ 0.633 GPS5/0.722 GPS6/0.722 GPS7/0.632 GPS8/0.683 GPS9/0.792
	Will and personality (WIP)	GPS10/0.764 GPS11/0.423
Social function (SOD)	Interpersonal communication (INC)	GSO1/0.782 GSO2/0.309 GSO3/0.350
	Social support and security (SSS)	GSO4/0.751 GSO5/0.812 GSO6/0.317
	Social role (SOR)	GSO7/0.261 GSO8/0.574

*Standardized path coefficients among domains and from domains to facets and items also see Fig. 2 in-detail.

**Figure 3:**
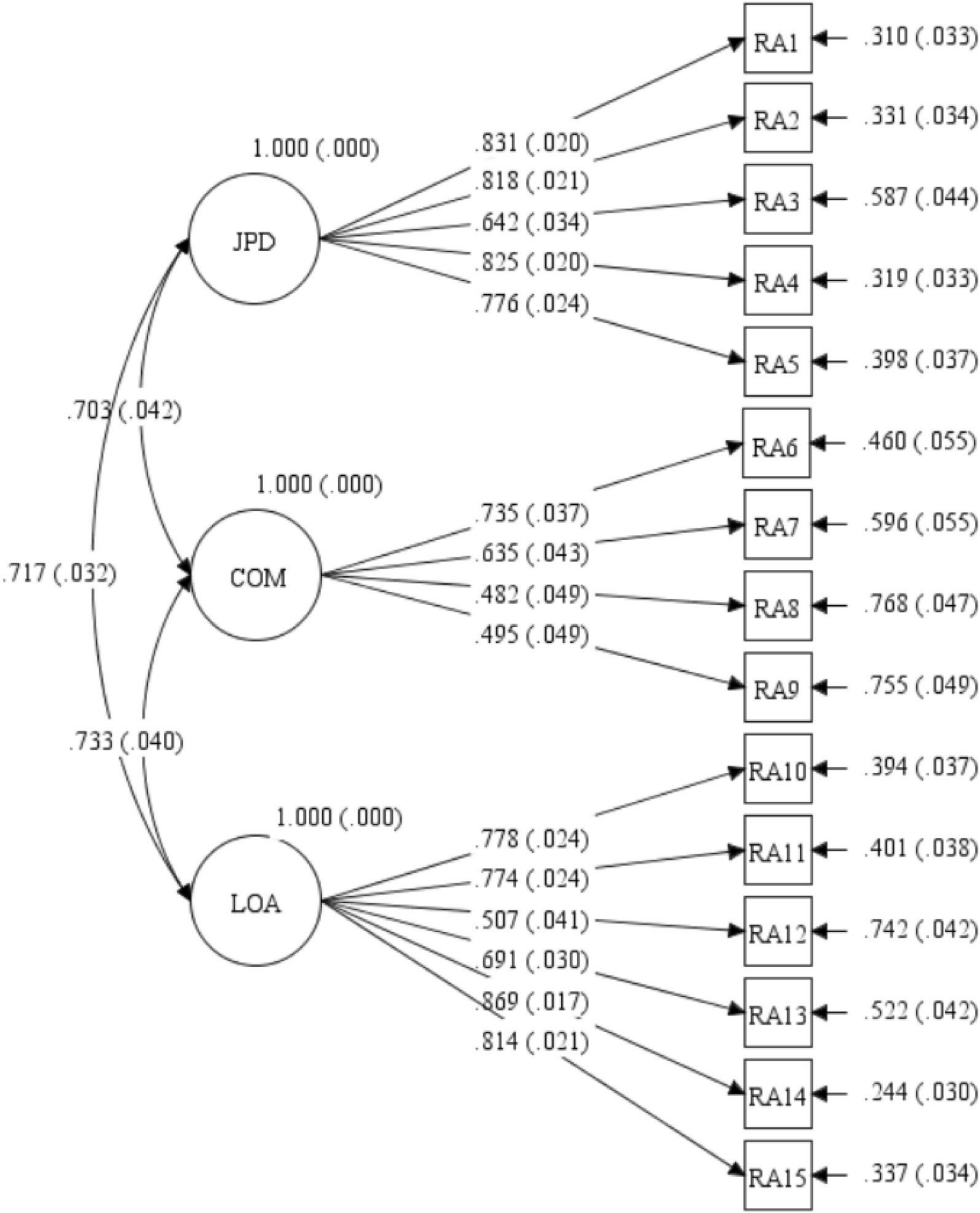
The structure of the specific module of QLICD-RA by structural equation modeling.

Structural equation modeling showed that the structure of the specific module of the QLICD-RA was consistent with the conceptual theoretical construct (three facets), with goodness of fit Chi-square ${{\varvec{\chi}}}^{2}$= 268.393(*P* < 0.001), df = 84, ${{\varvec{\chi}}}^{2}$/df = 3.195, Tucker–Lewis index (TLI) = 0.927, comparative fit index (CFI) = 0.942, root mean square error of approximation (RMSEA) = 0.076, standardized root mean square residual (SRMR) = 0.056. See Table 5 and Fig. 3 in detail.

**Table 5:** The results of SEM analysis on thespecific module of QLICD-RA (*n* = 379)*.

Facets	Items	Path coefficients	SE	Z	P	Standardized path coefficients
JPD (Joint pain and deformity)	RA1	1.000	0.000			0.831
	RA2	1.046	0.056	18.807	< 0.001	0.818
	RA3	0.856	0.066	12.889	< 0.001	0.642
	RA4	0.987	0.053	18.583	< 0.001	0.825
	RA5	0.885	0.053	16.722	< 0.001	0.776
COM (complication)	RA6	1.000	0.000			0.735
	RA7	0.813	0.089	9.144	< 0.001	0.635
	RA8	0.540	0.073	7.443	< 0.001	0.482
	RA9	0.546	0.074	7.336	< 0.001	0.495
LOA (Limitation of activity)	RA10	1.000	0.000			0.778
	RA11	0.993	0.048	20.866	< 0.001	0.774
	RA12	0.628	0.064	9.800	< 0.001	0.507
	RA13	0.897	0.066	13.628	< 0.001	0.691
	RA14	1.141	0.065	17.682	< 0.001	0.869
	RA15	1.023	0.061	16.778	< 0.001	0.814

*Standardized path coefficients among facets and from facets to items also see Fig. 3 in-detail.

#### Criteria-related validity

Table 6 shows the Pearson correlation coefficients between the physical, psychological, social and specific domains of QLICD-RA with the eight domains (or subscales) of SF-36. It can be seen that the correlations between the same and similar domains are generally higher than those between different and non-similar domains. For example, the correlation coefficient between the physical domain of QLICD-RA and the physical function domain of SF-36 (r = 0.71) is higher than that with other domains of SF-36. The higher correlation coefficients are also seen between psychological domain of QLICD-RA and mental health domain of SF-36 (r = 0.61); social domain of QLICD-RA and Social function of SF-36 (r = 0.51).

**Table 6:** Correlation coefficients among domains scores of QLICD-RA(V2.0) and SF-36 (n = 379).

QLICD-RA	SF-36							
	Physical function	Role-physical	Body pain	General health	Vitality	Social function	Role- emotional	Mental health
Physical domain	0.71	0.66	0.47	0.58	0.60	0.60	0.47	0.42
Psychological domain	0.34	0.42	0.26	0.46	0.51	0.44	0.41	0.61
Social domain	0.50	0.45	0.39	0.51	0.55	0.51	0.37	0.46
The specific module	0.62	0.65	0.39	0.47	0.51	0.53	0.36	0.44

Correlations in bold were that for similar domains.

#### Responsiveness

There were 223 patients who completed the questionnaire at the third assessment in order to evaluate the responsiveness. As shown in Table 7, more than half of the facets (seven out of 12) are seen with significant differences in scores before and after treatment (*p* < 0.05). In addition, the changes in scores of the physical and social domain of the general module are significant (*p* = 0.027 and *p* = 0.001, respectively). The value of SRM ranges from 0.07 to 0.27 for significant domains/facets, with the largest SRM being the facet of ‘Joint pain and deformity’ in the specific module of the instrument.

**Table 7:** Responsiveness of the quality of life instrument QLICD-RA(V2.0) (n = 223).

QLICD-RA	Before treatment		After treatment		Differences		*t*	*p*	SRM
	Mean	SD	Mean	SD	Mean	SD			
Physical domain	**56.34**	**17.21**	**58.00**	**16.32**	**1.88**	**22.78**	**− 2.222**	**0.027**	**0.073**
Basic physiologic functions	55.77	15.84	55.72	14.90	**− **0.06	11.94	0.07	0.944	0.005
Independence	65.36	29.12	67.00	27.08	1.64	18.83	**− **1.304	0.194	0.087
Energy and discomfort	43.95	23.49	49.05	24.14	5.10	22.42	**− **3.398	0.001	0.228
Psychological domain	**58.15**	**17.79**	**59.25**	**18.88**	**1.10**	**12.40**	**− 1.325**	**0.186**	**0.089**
Cognition	61.21	20.73	61.27	21.68	0.06	17.62	**− **0.048	0.962	0.003
Emotion	55.22	20.48	57.98	20.92	2.75	14.60	**− **2.817	0.005	0.189
Will and personality	65.36	21.35	61.72	21.41	**− **3.64	18.46	2.948	0.004	0.197
Social domain	**69.83**	**16.35**	**67.54**	**15.90**	**− 2.28**	**9.89**	**3.449**	**0.001**	**0.231**
Interpersonal communication	76.79	16.70	74.03	16.79	**− **2.77	12.65	3.265	0.001	0.219
Social support and security	68.46	19.45	65.66	18.56	**− **2.80	13.65	3.066	0.002	0.205
Social role	61.44	23.99	60.65	22.42	**− **0.78	19.22	0.61	0.543	0.041
Sub-total (QLICD-GM)	**60.91**	**14.32**	**61.22**	**14.95**	**0.31**	**8.55**	**− 0.545**	**0.586**	**0.037**
Specific domain	**63.12**	**20.73**	**64.72**	**20.43**	**1.61**	**13.99**	**− 1.715**	**0.088**	**0.115**
Limitation of activity	69.82	25.80	71.06	24.41	1.23	18.84	**− **0.978	0.329	0.065
Complications & side effects	70.63	19.46	68.30	20.95	2.33	16.13	2.15	0.032	0.144
Joint pain and deformity	49.06	25.27	54.26	24.12	5.20	19.49	**− **3.985	< 0.001	0.267
Total (TOT)	**61.68**	**15.08**	**62.44**	**15.55**	**0.76**	**8.88**	**− 1.284**	**0.200**	**0.086**

The values at domain/overall level are in bold.

## Discussions

This paper focused on the development and validation of the QLICD-RA (V2.0), a specific QOL instrument for Rheumatoid Arthritis Scale among the System of Quality of Life Instruments for Chronic Diseases. It demonstrated good psychometric properties in terms of reliability and validity in Chinese speaking adult RA patients.

In terms of reliability, internal consistency reliability (Cronbach’s α), test–retest reliability (Pearson r) and ICC were applied in the current study. All domains and the overall score of the QLICD-RA demonstrated excellent internal consistency by a relatively high Cronbach’s α (range 0.77–0.94), indicating that all items are measuring the same thing. The subscale/domain scores of QLICD-RA had high test–retest reliability (both Pearson r and ICC ranged from 0.86 to 0.99) according to the correlation between the first- and second-time measurements. Thus this instrument has excellent reliability considering that internal consistency coefficients above 0.70 and test–retest reliability coefficient above 0.80 are generally accepted as satisfactory.

The duration of these two measurements is one day, which may result in memory effect. In the previous studies, the duration between test and retest is normally 14 days or four weeks for healthy people^17,37,38^. Given some practical factors, such as the relatively short duration of admission in hospital, the potential (“quick”) changes in QOL caused by the therapy and the discussion by expert panel, we decided to conduct the retest measure one day after the first measurement. According to our experiences^25,27^, one-day interval is stable and not much more memory effect because too many items and the patients do not know it will repeat again.

The present study demonstrates a good validity of QLICD-RA. More specifically, our data supports a good convergent and discriminant validity, because all the item-domain correlation coefficients are greater than 0.4, and all the correlations between items and designated domains are higher than that between items and non-designated domains, except for the item GPS1 (‘Attention’). The item GPS1 is “Can you focus attention on what you are doing?” The correlation between GPS1 and physical/social domain (i.e. 0.57 and 0.49) is higher than that with psychological domain (i.e. 0.42), which is not consistent with our hypothesis. This finding may be related to how patients perceived and understood this item. Probably, from perspectives of patients, the attention issue was more related to their physical and social health status due to living with RA than psychological health. We suggested to ask the RA patients in the further study regarding how they perceive the wording of this item and maybe in the future we will rephrase this item.

Moreover, the validity of the construct was also further confirmed by structural equation modeling, which revealed excellent fit for the specific module from the data corresponded with the theoretical constructs of the instrument^35,36^. In contrast, it just shows basically acceptable fit for the general module in RA data in this research. However, the general module can be used for all patients with chronic diseases, and has been confirmed excellent fit by SEM from 11 diseases data (report elsewhere). Therefore, it can be confirmed that the general module of the QLICD-RA was consistent with the conceptual theoretical construct (three domains, nine facets), although SEM in RA data has only just basically acceptable fit.

With regard to the criteria-related validity, as expected, our study shows a good correlation between physical domain of QLICD-RA and physical function domain of SF-36; as well as psychological domain of QLICD-RA and mental health domain of SF-36. However, the social domain of QLICD-RA was significantly correlated with the social function domain (0.51) but also the vitality domain (0.55) of SF-36. The specific module score is highly significantly correlated with the Physical function and role-physical domain of SF-36. This finding is consistent with the nature of question items in both domains. The questions of specific module are mainly about the discomforting symptoms and the physical limitations due to RA. Besides, these correlation coefficients also revealed the convergent and divergent validity to some extent, which again confirmed the good construct validity.

With regard to the responsiveness, the assessment methods on responsiveness can be divided into two categories: internal and external^39,40^. In this paper we focused on the internal responsiveness with the hypothesis that the sensitive instrument should detect changes in response to treatments when assessed at post-treatment. We did not find the significant change in the scores of overall instrument, generic/specific module and the psychological domain before and after treatment. We found the significant changes regarding the physical and social domain scores, i.e. the physical domain score has been increased after treatment and the social domain score decreased. This finding could be explained by that the treatment in hospital probably has relieved the discomforting symptoms and improved patient’s physical health, while the admission in hospital may limit the social functioning of the patient. The above explanation could also be applied to the change in the scores in specific facets, such as “Energy and discomfort”, “interpersonal communication”, “social support and security”, “social roles”, “treatment side effects” and “joint pain and deformity”.

To our best knowledge, the present study is the first study in China to validate QLICD-RA (V2.0) in the clinical patients with RA in a relatively large sample. We have evaluated a comprehensive set of the psychometric parameters, including reliability, validity and responsiveness, which provided the valuable evidence for the clinical professionals to apply this instrument in the clinical research and daily practice. The QLICD-RA has several advantages over existing instruments. First, it could compare QOL across diseases by the general module and also capture the symptoms and side effects by the specific module. Second, it is of the strong Chinese cultural background. For example, the Chinese culture pay more attention to family relationship and kinship, dietary, temperament and high spirit, which are all captured in the QLICD-RA by items focusing on appetite (GPH1), sleep (GPH2), energy (GPH9) and family support (GSO2, GSO4 etc.).

However, there are several limitations that warrant attention. First, the sample to evaluate the test–retest reliability is relatively small, and the internal between both tests is relatively short. Second, though we have reduced the amount of items based on several rounds of expert panels and pretest, the total number of items of the second version QLICD is 43, which may cause respond burden for patients in certain circumstances. We recommend the future research to carefully assess the time spent on filling in the questionnaire and the barriers to understand and complete it. Third, given the composition of the sample in our study, patients were more often from relatively low socio-economic status, which may limit the generalization of our study. And all the patients are inpatients in our study. We suggest to duplicate the validation of QLICD-RA in a more representative population, in the outpatient population, and in other geographic areas in China where the socio-cultural characteristics may be different from the area where the patients in our study resided, and may influence the psychometric performance of this instrument.

## Conclusions

Our study shows that the second version of QLICD-RA has a good reliability, validity and responsiveness. It can be used to measure QOL among patients with RA in mainland China. Other foreign language versions can develop rigorous translation programs based on this scale. We suggest the future studies to duplicate the present study in other settings, such as RA outpatients in hospital, a population with different socio-demographic background, to extend the evidence pool in terms of the validation of this instrument.

## Data Availability

The datasets used and/or analysed during the current study available from the corresponding author on reasonable request.

## Abbreviations

RA: Rheumatoid arthritis
QLICD-RA: Quality of Life Instruments for Chronic Diseases- Rheumatoid arthritis
CTT: Classical test theory
QoL: Quality of life
ICC: Intra-class correlation
SRM: Standardized response mean
CSHQ-RA: Cedars-Sinai Heath-Related Quality of Life in Rheumatoid Arthritis instrument
PKQ: Patient Knowledge Questionnaire
eCQMs: Electronic Clinical Quality Measures
ACREU: Arthritis Community Research and Evaluation Unit
AIC: The Arthritis information course
AIMS: The Arthritis Impact Measurement Scales
RAQOL: The Rheumatoid Arthritis Quality of life Scale

## References

[1] World Health Organization. Chronic rheumatic conditions. Retrieved from 7 February 2020. https://www.who.int/chp/topics/rheumatic/en/

[2] Hansen SM, Hetland ML, Pedersen J (2017). Work ability in rheumatoid arthritis patients: A register study on the prospective risk of exclusion and probability of returning to work. Rheumatology.

[3] Malm K, Bergman S, Andersson MLE (2017). Quality of life in patients with established rheumatoid arthritis: A phenomenographic study. SAGE Open Med..

[4] Ometto F, Fedeli U, Schievano E (2018). Cause-specific mortality in a large population-based cohort of patients with rheumatoid arthritis in Italy. Clin. Exp. Rheumatol..

[5] Zeng XF, Zhu SL, Tan AC (2013). Disease burden and quality of life of rheumatoid arthritis in China: A systematic review. Chin. J. Evid. Based Med..

[6] Xu C, Wang X, Mu R (2014). Societal costs of rheumatoid arthritis in China: A hospital-based cross-sectional study. Arthr. Care Res..

[7] The World Health Organization Quality of Life assessment (WHOQOL): Position paper from the World Health Organization. (1995), Soc. Sci. Med, 41(10), 1403–9 10.1016/0277-9536(95)00112-k 8560308

[8] De Jong Z, Van der Heijde D, McKenna SP (1997). The reliability and construct validity of the RAQoL: A rheumatoid arthritis-specific quality of life instrument. Br. J. Rheumatol..

[9] Lubeck DP (2002). Health-related quality of life measurements and studies in rheumatoid arthritis. Am. J. Manag. Care.

[10] Larice S, Ghiggia A, Di Tella M (2019). Pain appraisal and quality of life in 108 outpatients with rheumatoid arthritis. Scand. J. Psychol..

[11] Berner C, Erlacher L, Quittan M (2017). Workability and muscle strength in patients with seropositive rheumatoid arthritis: Survey study protocol. JMIR Res. Protoc..

[12] Wolfe F (2001). Which HAQ is best? A comparison of the HAQ, MHAQ and RA-HAQ, a difficult 8 item HAQ (DHAQ), and a rescored 20 item HAQ (HAQ20): Analyses in 2,491 rheumatoid arthritis patients following leflunomide initiation. J. Rheumatol.

[13] Brunner HI, Johnson AL, Barron AC (2005). Gastrointestinal symptoms and their association with health-related quality of life of children with juvenile rheumatoid arthritis: Validation of a gastrointestinal symptom questionnaire. JCR J. Clin. Rheumatol..

[14] Danao LL, Padilla GV, Johnson DA (2001). An English and Spanish quality of life measure for rheumatoid arthritis. Arthr. Care Res..

[15] Whalley D, McKenna SP, De Jong Z (1997). Quality of life in rheumatoid arthritis. Br. J. Rheumatol..

[16] Russak SM, Sherbourne CD, Lubeck DP (2003). Validation of a rheumatoid arthritis health-related quality of life instrument, the CSHQ-RA. Arthr. Care Res.: Off. J. Am. Coll. Rheumatol..

[17] Chiou CF, Sherbourne CD, Cornelio I (2006). Development and validation of the revised Cedars-Sinai health-related quality of life for rheumatoid arthritis instrument. Arthr. Care Res.: Off. J. Am. Coll. Rheumatol..

[18] Duffy C, Arsenault L, Duffy K (1997). The Juvenile Arthritis Quality of Life Questionnaire-development of a new responsive index for juvenile rheumatoid arthritis and juvenile spondyloarthritides. J. Rheumatol..

[19] Meenan RF, Mason JH, Anderson JJ (1992). AIMS2: The content and properties of a revised and expanded arthritis impact measurement scales health status questionnaire. Arthr. Rheumatol..

[20] Yilmaz V, Umay E, Aras B (2019). Health-related quality of life outcomes of young adults with juvenile idiopathic arthritis in turkish population. SN Compr. Clin. Med..

[21] Tijhuis GJ, De Jong Z, Zwinderman AH (2001). The validity of the rheumatoid arthritis quality of life (RAQoL) questionnaire. Rheumatology.

[22] Hedin PJ, McKenna SP, Meads DM (2006). The rheumatoid arthritis quality of life (RAQoL) for Sweden: Adaptation and validation. Scand. J. Rheumatol..

[23] Aaronson NK, Cull A, Kaasa S (1994). The European Organization for Research and Treatment of Cancer (EORTC) modular approach to quality of life assessment in oncology. Int. J. Ment. Health.

[24] Cella D, Nowinski CJ (2002). Measuring quality of life in chronic illness: The functional assessment of chronic illness therapy measurement system. Arch. Phys. Med. Rehabilit..

[25] Wan C, Tu X, Messing S (2011). Development and validation of the general module of the system of quality of life instruments for chronic diseases and its comparison with SF-36. J. Pain Symptom Manage..

[26] Wan CH, Li XM, Yang Z (2019). Development and applications of the system of quality of life instruments for chronic diseases QLICD(V10) (in Chinese). China Sci. Technol. Achiev..

[27] Liu Y, Chang Y, Wan D (2023). Development and validation of a disease-specific quality of life measure QLICD-HY (V2.0) for patients with hypertension. Sci. Rep..

[28] Liu Q, Feng L, Wan C (2022). Development and validation of the psoriasis scale among the system of quality of life instruments for chronic diseases QLICD-PS (V2.0). Health Qual. Life Outcomes.

[29] Quan P, Yu L, Yang Z, Lei P, Wan C, Chen Y (2018). Development and validation of quality of life instruments for chronic diseases-Chronic gastritis version 2 (QLICD-CG V2.0). PLoS One.

[30] Li F, Liu Y, Wan C (2022). Establishing minimal clinically important differences for the quality of life instrument in patients with breast cancer QLICP-BR (V2.0) based on anchor-based and distribution-based methods. Front Oncol.

[31] Ma Z, Liu Y, Wan C (2022). Health-related quality of life and influencing factors in drug addicts based on the scale QLICD-DA: A cross-sectional study. Health Qual. Life Outcomes.

[32] Yang Z, Li W, Tu XM (2012). Validation and psychometric properties of chinese version of SF-36 in patients with hypertension, coronary heart diseases, chronic gastritis and peptic ulcer. Int. J. Clin. Pract..

[33] Henson RK (2001). Understanding internal consistency reliability estimates: A conceptual primer on coefficient alpha. Meas. Eval. Couns. Dev..

[34] Hays RD, Hayashi T (1990). Beyond internal consistency reliability: Rationale and use’s guide for multi-trait analysis program on the microcomputer. Behav. Res. Methods Instrum. Comput..

[35] Hu LT, Bentler P (1999). Cutoff criteria for fit indexes in covariance structure analysis: Conventional criteria versus new alternatives. Struct. Equ. Model..

[36] Marsh HW, Hau KT, Wen Z (2004). In search of golden rules: Comment on hypothesis-testing approaches to setting cutoff values for Fit indexes and dangers in overgeneralizing Hu and Bentler's (1999) findings. Struct. Equ. Model.: Multidiscip. J..

[37] Islam N, Khan IH, Ferdous N (2017). Translation, cultural adaptation and validation of the English “Short form SF 12v2” into Bengali in rheumatoid arthritis patients. Health Qual. Life Outcomes.

[38] Nadrian H, Niaz YH, Basiri Z (2019). Development and psychometric properties of a self-care behaviors scale (SCBS) among patients with rheumatoid arthritis. BMC Rheumatol..

[39] Husted JA, Cook RJ, Farewell VT, Gladman DD (2000). Methods for assessing responsiveness: A critical review and recommendations. J. Clin. Epidemiol..

[40] Terwee CB, Dekker FW, Wiersinga WM (2003). On assessing responsiveness of health-related quality of life instruments: Guidelines for instrument evaluation. Qual. Life Res..

